# Supplementary material for: Cortisol Awakening Reaction and Anxiety in Depressed Coronary Artery Disease Patients
Source: J Clin Med. 2022 Jan 13;11(2):374. doi: 10.3390/jcm11020374 (PMC8779785; doi:10.3390/jcm11020374)
Supplement: Supplementary file 1 [file jcm-11-00374-s001.zip › jcm-1507226-supplementary.pdf]

# Supplementary Material

**Table S1.** Correlation analyses.

| Variables | AUCg | | AUCi | |
|---|---|---|---|---|
| | r | *p* | r | *p* |
| Age | 0.108 | 0.348 | −0.129 | 0.264 |
| BMI | −0.028 | 0.814 | −0.081 | 0.490 |
| Sex | −0.195 | 0.089 | 0.071 | 0.537 |
| Beta blocker | −0.072 | 0.536 | −0.043 | 0.710 |
| CCI | −0.089 | 0.444 | −0.147 | 0.201 |
| Number of affected vessels | −0.029 | 0.808 | 0.134 | 0.253 |
| LVEF | −0.279 | 0.032 | −0.163 | 0.217 |
| HADS depression | 0.079 | 0.493 | 0.195 | 0.089 |
| HADS anxiety | <0.001 | 0.999 | 0.218 | 0.057 |
| PHQ depression | 0.042 | 0.726 | 0.117 | 0.325 |
| DS-14 negative affectivity | 0.132 | 0.254 | −0.031 | 0.789 |
| DS-14 social inhibition | 0.021 | 0.854 | −0.012 | 0.919 |
| MQ vital exhaustion | 0.076 | 0.525 | 0.170 | 0.150 |
| SF-36 physical health sum score | 0.017 | 0.886 | 0.001 | 0.991 |
| SF-36 mental health sum score | −0.072 | 0.553 | −0.083 | 0.492 |

**Table S2.** Repeated-measures ANOVA 4 steps (cortisol +0, +30, +45, +60), 2 groups (anxious vs. non anxious).

| Source of variation | Type III Sum of Squares | Mean Square | F | df | *p* |
|---|---|---|---|---|---|
| Time | 670.613 | 286.126 | 9.003 | 2.344 | <0.001 |
| Time x Group | 398.871 | 170.184 | 5.355 | 2.344 | 0.003 |

**Table S3.** Repeated-measures ANOVA 4 steps (cortisol +0, +30, +45, +60), 2 groups (anxious vs. non anxious) with confounders (LVEF, hyperlipidemia).

| Source of variation | Type III Sum of Squares | Mean Square | F | df | *p* |
|---|---|---|---|---|---|
| Time | 334.017 | 148.322 | 4.564 | 2.252 | 0.009 |
| Time x LVEF | 161.848 | 71.869 | 2.211 | 2.252 | 0.107 |
| TIME x hyperlipidemia | 53.447 | 23.733 | 0.730 | 2.252 | 0.499 |
| Time x Group | 407.902 | 181.131 | 5.573 | 2.252 | 0.003 |

**Table S4.** Repeated-measures ANOVA 4 steps (cortisol +0, +30, +45, +60), 2 groups (anxious vs. non anxious) with confounders (smoking status, Type D, SES).

| Source of variation | Type III Sum of Squares | Mean Square | F | df | *p* |
|---|---|---|---|---|---|
| Time | 265.786 | 114.875 | 3.724 | 2.314 | 0.021 |
| Smoking status | 141.306 | 61.074 | 1.980 | 2.314 | 0.134 |

| | | | | | |
|---|---|---|---|---|---|
| Type D | 97.613 | 42.189 | 1.368 | 2.314 | 0.258 |
| SES | 174.055 | 75.228 | 2.439 | 2.314 | 0.082 |
| Time x Group | 631.708 | 273.029 | 8.850 | 2.314 | <0.001 |

**Table S5.** Repeated-measures ANOVA 4 steps (cortisol +0, +30, +45, +60), 2 groups (anxious vs. non anxious) with confounders (age, sex).

| **Source of variation** | **Type III Sum of Squares** | **Mean Square** | **F** | **df** | ***p*** |
|---|---|---|---|---|---|
| Time | 30.704 | 13.101 | 0.404 | 2.344 | 0.701 |
| Age | 17.180 | 7.330 | 0.226 | 2.344 | 0.831 |
| Sex | 21.762 | 9.285 | 0.286 | 2.344 | 0.785 |
| Time x Group | 338.626 | 144.487 | 4.454 | 2.344 | 0.009 |
